# Exploring New Antioxidant and Mineral Compounds from *Nymphaea alba* Wild-Grown in Danube Delta Biosphere

**DOI:** 10.3390/molecules23061247

**Published:** 2018-05-23

**Authors:** Mihaela Cudalbeanu, Ioana Otilia Ghinea, Bianca Furdui, Durand Dah-Nouvlessounon, Robert Raclea, Teodor Costache, Iulia Elena Cucolea, Florentina Urlan, Rodica Mihaela Dinica

**Affiliations:** 1 Faculty of Sciences and Environment, Department of Chemistry Physical and Environment, “Dunarea de Jos” University of Galati, 111 Domneasca Street, 800201 Galati, Romania; Mihaela.Cudalbeanu@ugal.ro (M.C.); Ioana.Ghinea@ugal.ro (I.O.G.); bfurdui@ugal.ro (B.F.); 2 Faculty of Sciences and Techniques, Department of Biochemistry and Cell Biology, Laboratory of Biology and Molecular Typing in Microbiology, University of Abomey-Calavi, Cotonou 05BP1604, Benin; dahdurand@gmail.com; 3 Imperial College London, Faculty of Natural Sciences, Department of Chemistry, London SW7 2AZ, UK; robert.raclea@yahoo.com; 4 Research Center for Instrumental Analysis SCIENT, 1E Petre Ispirescu Street, 077167 Tancabesti, Ilfov, Romania; teodor.costache@scient.ro (T.C.); iulia.cucolea@scient.ro (I.E.C.); florentinaurlan@yahoo.com (F.U.)

**Keywords:** *Nymphaea alba* extracts, antioxidant compounds, Danube Delta Biosphere

## Abstract

*Nymphaea alba* is an aquatic flowering plant from the *Nymphaeaceae* family that has been used for hundreds of years in traditional herbal medicine. The plant is characterized by different phytochemicals, depending on the geographical location. Herein, we have carried out, for the first time, the separation and HPLC-MS/MS identification of some antioxidant compounds, such as polyphenols and flavonoids from *N. alba* extracts from the Danube Delta Biosphere, and investigated their possible antiradical properties. An ultrasonic method has been exhaustively used for the extraction of the antioxidant compounds from the different anatomic parts of *N. alba* (fruit, flower, leaf, stem, and root). The extracts that were obtained using ultrasound irradiation showed a large polyphenol (19.42 mg EqGA/100 mg extract) and flavonoid (0.97 mg EqQ/100 mg extract) content. The fruit and flower extracts showed the highest antioxidant activity index (AAI). Among the 27 phytochemical compounds identified in all of the *N. alba* extracts, rutin and p-coumaric acid were found as the major components. The content of macroelements and microelements in *N. alba* extracts were compared, and it was found that their concentrations depend on the different anatomic parts of the plant. This research contributes to the study of *Nymphaeaceae* family, being the first exhaustive phytochemical study of *N. alba* from a wild population in Romania.

## 1. Introduction

*Nymphaea alba* species (*N. alba*), also known as the European white water lily, belongs to the *Nymphaea* genus and the *Nymphaeaceae* family [1,2]. Species from the *Nymphaeaceae* family, such as *N. alba*, have important medicinal properties, being used in the treatment of diabetes, inflammations, liver disease, urinary tract infection, and they also have tonic and aphrodisiac properties [3]. Previous studies have reported the antioxidant, anti-inflammatory, and hepatoprotective effects of the *N. alba* species. These effects can be attributed to some phytochemical components from the polyphenol class, such as ellagic acid, gallic acid, and their methyl and ethyl esters, in addition to flavonoids such as quercetin or kaempferol [2,4].

Polyphenols and flavonoids are the largest group of phytochemical compounds and are omnipresent secondary metabolites amongst plants. From ancient times, people have used plants for infectious diseases, and scientific research has proved their therapeutic effects over time [5]. Recent research supports the role of these types of secondary metabolites in the prevention of degenerative diseases, especially cancer, cardiovascular diseases, and neurodegenerative diseases [6]. Polyphenols and flavonoids are strong antioxidants that complete and add to the functionalities of vitamins and antioxidant enzymes with the purpose of defense against oxidative stress caused by the excessive presence of reactive oxygen species (ROS) [7,8]. Some compounds, such as gallic acid, have a strong antioxidant activity, while others, such as monophenols, are weak antioxidants [9]. In vacuoles, flavonoids are found either in a free state or bound to carbohydrates (glucose, galactose, rhamnose, mannose, etc.), and they tend to be soluble in water or organic solvents [10,11]. Natural antioxidants from plants play an important role in maintaining general health [12], and they have garnered interest amongst consumers and scientific communities alike, since epidemiologic studies have shown that the frequent consumption of natural antioxidants is associated with a lowered risk of chronic or degenerative cardiovascular disease, such as arthrosclerosis, cardiac and cerebral ischemia, carcinogenesis, neurogenerative diseases, gestational diabetes, rheumatic disease, DNA deterioration, and skin aging [9,11], as well as infections of the respiratory, urinary, and gastrointestinal tract, and the biliary system [13].

In Romania, the natural habitats of *Nymphaea alba* are found in the Danube Delta landscape, which shelters a broad large of water macrophyte communities. This study is the first detailed chemical investigation that has been exhaustively done over all of the anatomic parts of *N. alba* from the Danube Delta Biosphere Reserve; it includes ultrasound irradiation as a method of extraction, and the identification and characterization of the phytochemicals through chromatographic techniques, such as LC-MS/MS and inductively coupled plasma optical emission spectrometer (ICP OES) technique. The beneficial effects of the plants that possess medicinal properties are influenced also by inorganic ingredients, so it is important to know their levels of macroelements and microelements. A correlation between flavonoid and polyphenol content with the antioxidant activity of the methanolic extracts of *N. alba* was also illustrated. To the best of our knowledge, this is the first phytochemical study of *N. alba* from a wild population in Romania.

## 2. Results and Discussion

### 2.1. Total Polyphenol Content

**Figure 1:**
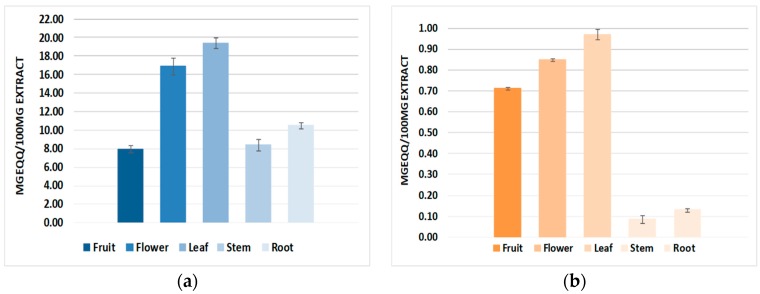
Total concentration of polyphenols (**a**) and flavonoids (**b**) determined from the *N. alba* methanolic fruit, flower, leaf, stem, and root extracts.

The total polyphenol content was determined spectrophotometrically using gallic acid as the calibration standard for the *N. alba* fruit, flower, leaf, stem, and root extracts obtained by ultrasonic extraction. The total polyphenol content showed a large variance, ranging from 7.95 to 19.42 mg EqGA/100 mg of extract. The largest polyphenol quantities were found in the leaf extract (19.42 mg EqGA/100 mg of extract) and flower extract (16.90 mg EqGA/100 mg of extract) (Figure 1a). Literature data about another *Nymphaea* species [2,14,15] confirms the total polyphenol content values for our *N. alba* extracts.

### 2.2. Total Flavonoid Content

Following the LC-MS/MS analyses conducted on the *N. alba* extracts, the presence of flavonoids such as quercetin, catechin, epicatechin, naringenin, and naringin was confirmed. The total flavonoid content was spectrophotometrically quantified using quercetin as a calibration standard. The total flavonoid content of the *N. alba* fruit, flower, leaf, stem, and root extracts obtained by ultrasonic extraction was determined. The values ranged from 0.09 to 0.97 mg EqQ/100 mg of extract. The largest flavonoid amount is present in the leaf extract, with a value of 0.97 mgEqQ/100 mg of extract, and in the flower extract, with a value of 0.85 mg EqQ/100 mg of extract (Figure 1b).

### 2.3. Condensed Tannins Content

Condensed tannins are of interest because of their antioxidant activity and other potentially beneficial effects for human health. Nevertheless, their biological activity depends on the structure and their concentration [16,17]. The total condensed tannins content for the different anatomic parts of *N. alba* from the Danube Delta Biosphere Reserve was determined for the first time spectrophotometrically using catechin as the calibration standard. The total content varied between 0.06–2.87 mg EqC/g. Condensed tannins are present in the methanolic fruit, leaf, stem, and root extracts, data which is proven also by LC-MS/MS chromatograms. The largest content of condensed tannins is present in the methanolic *N. alba* stem extract, with a value of 2.87 mg EqC/g (Table 1).

### 2.4. Antioxidant Activity

**Figure 2:**
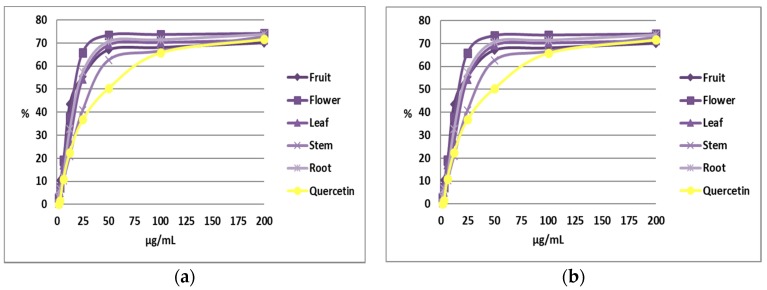
DPPH inhibition percentage of the methanolic *N. alba* fruit, flower, leaf, stem, and root extracts after 20 min of incubation (**a**); and after 50 min of incubation (**b**).

Natural antioxidants protect cells by the elimination of very reactive free radicals and ROS from biological systems [11]. The antioxidant activity has been measured for the methanolic *N. alba* extracts obtained by ultrasonic extraction. The IC_50_ value represents the summed concentration of compounds that are necessary in order to reduce the initial DPPH concentration by 50%. IC_50_ values for the methanolic extracts have been determined using a graphical representation of the percentage of inhibition versus concentration. The biggest antioxidant activity was found in the methanolic flower extract. The IC_50_ values for the methanolic *N. alba* extracts are as follows: fruit- (17 µg/mL), flower (17 µg/mL), leaf (20 µg/mL), stem (25 µg/mL), and root (19 µg/mL) (Figure 2a). The DPPH inhibition percentages for all of the *N. alba* extracts after 50 minutes of incubation prove that the antioxidant activity of these compounds is stable over time (Figure 2b).

**Figure 3:**
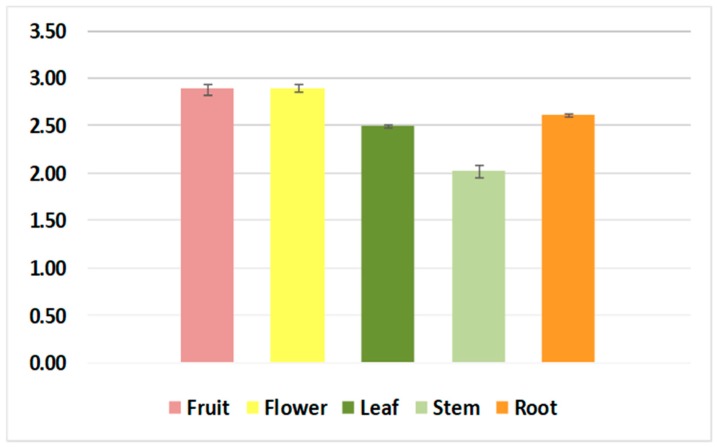
Antioxidant activity index (AAI) of the methanolic *N. alba* fruit, flower, leaf, stem, and root extracts.

The large-scale use of the DPPH method and the lack of standardization of the results make it difficult to compare the antioxidant resistances of different plant extracts and pure compounds. For this reason, an antioxidant activity index (AAI) has been determined for the DDPH method [18]. For plant extracts or pure compounds, data such as the percentage of DPPH inhibition of IC_50_ values modify as a function of the final concentration of used DPPH (Figure 3).

The antioxidant activity index (AAI) value indicates the success of a compound as an antioxidant. All of the tested extracts showed a strong antioxidant activity (AAI > 2). No significant differences were observed between the fruit and flower extracts, which showed higher AAI values than the leaf and root extracts, which were similar to each other. The stem extract showed the lowest AAI value. Fruit and flower extracts exhibited strong antioxidant activity, which was probably due to the presence of flavonoids as apigenin or luteolin, who had hydroxyl groups on the A ring, and could be involved in increasing the antioxidant potential of the extracts.

### 2.5. HPLC-MS/MS Identification of Polyphenolic Compounds

A new and sensitive method that combines high-performance liquid chromatography and ionization mass spectrometry for the separation, identification, and quantification of polyphenols, flavonoids, and tannins in the methanolic *N. alba* extracts has been adapted and optimized. Caffeic acid, chlorogenic acid, p-coumaric acid, catechin, epicatechin, naringin, naringenin, vanillic acid, quercetin, and rutin were used as reference standards. Sixteen MRM (Multiple Reaction Monitoring) experiments from the literature were also used: hexahydroxydiphenic axid (HHDP)-hexoside, corilagin, tannic acid, gallic acid, ferulic acid, ellagic acid, ellagic acid pentoside, quinic acid, kaempferol, castalin, orientin, apigenin, luteolin, brevifolin, ellagic acid, rhamnosyl, cinnamic acid derivatives, and another unidentified compound (Table 2).

The qualitative LC-MS/MS analysis of the methanolic *N. alba* extracts has shown a wide range of polyphenolic compounds. Using the reference standards and comparing the obtained mass spectra with the literature data has led to the identification of different fragments of polyphenolic compounds split into multiple compound subgroups: phenolic acids, flavonoids, tannins, and other non-flavonoid polyphenols. In total, 27 phytochemical compounds were identified in the methanolic *N. alba* extracts, and their retention time (T_R_), chemical formula, parent ion, and the fragment negative ion, relative abundance, and mass error are presented in Table 2.

#### Identification of Phenolic Compounds

**Figure 4:**
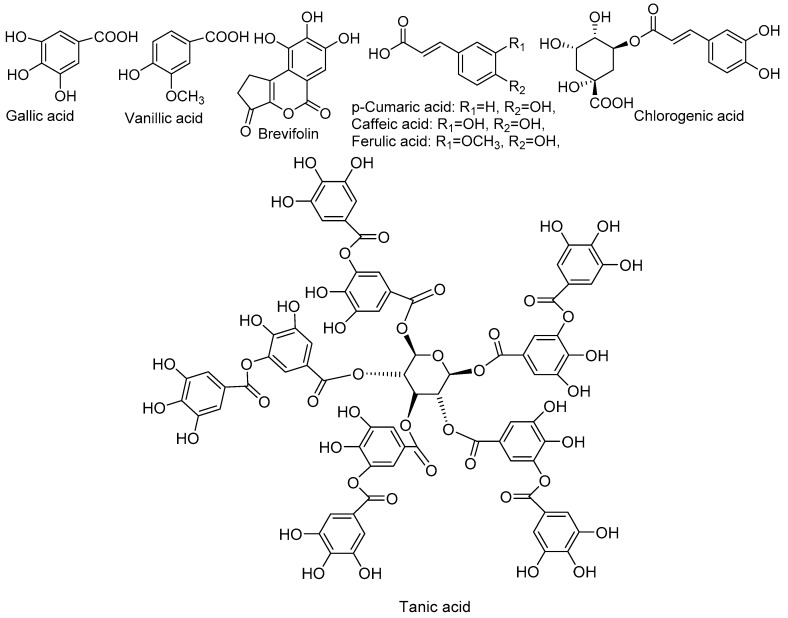
Structures of phenolic acids and derivatives.

*Phenolic Acids and Derivatives*. Compounds **4**, **7**, **10**, and **12** from chromatograms were identified as gallic, chlorogenic, caffeic, and tannic acid and other derivatives (Figure 4), by comparing their retention times and MS/MS profiles with those of the corresponding reference compounds. The deprotonated molecule ion at *m*/*z* 169 confirmed the presence of gallic acid.

**Figure 5:**
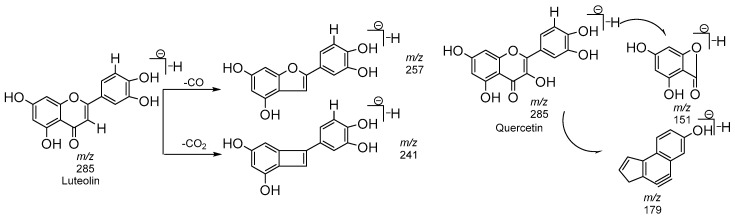
Fragmentation pathway of flavonoids.

**Figure 6:**
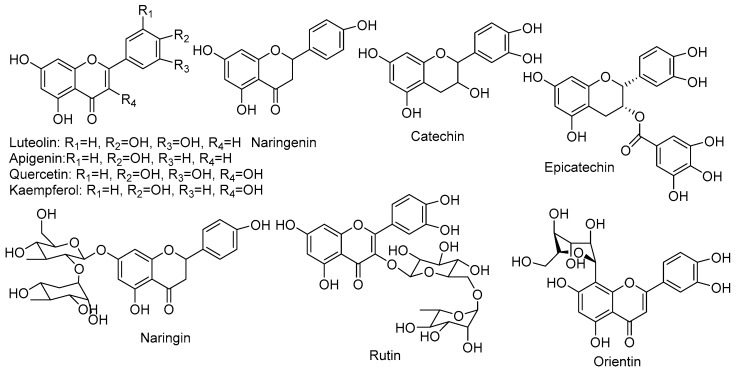
Structures of the main flavonoids compounds identified in *N. alba* extracts.

*Flavonoids*. Diagnostics mass fragments obtained at *m*/*z* 269, 285, and 301 characterized aglycones as apigenin, luteolin, kaempferol, quercetin (Figure 5), and naringenin, respectively (Figure 6), on the basis of comparing the retention time and the fragmentation pattern of the published data with those of an authentic sample (quercetin, naringenin). The values of *m*/*z* lower than the aglycone-like *m*/*z* 179 and 151 are typical of retro Dies–Alder reactions of flavan-3-ols (Figure 5) [24].

Compounds **24** and **25** correspond to catechin and epicatechin (Figure 6), respectively, as confirmed by comparing with standard and MS/MS product ions, which matched those of an authentic sample. Compound **13** was identified as rutin, whereas compound 19 was identified as naringin after comparison of its [M-H]^−^ and MS/MS product ions and retention time with those of an authentic standard. Compound **27** was identified as orientin, the luteolin-hexoside (Figure 6), on the basis of their characteristic [M-H]^−^ and MS/MS fragmentation profiles.

**Figure 7:**
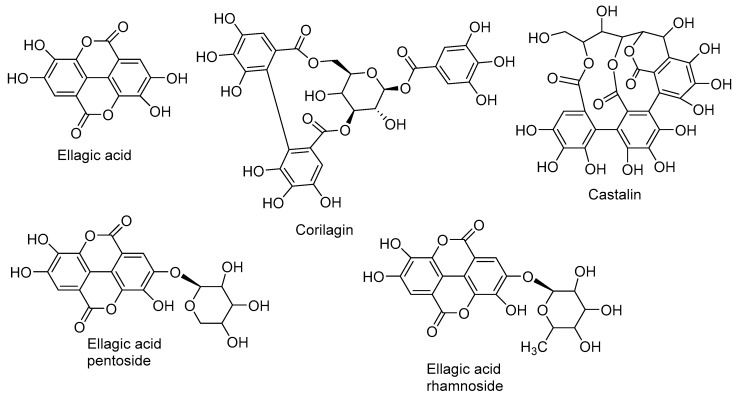
Structure of the main ellagic derivatives identified in *N. alba* extracts.

*Ellagic Acid and Derivatives.* Compound **14** (Figure 7) was identified as ellagic acid after comparison of its fragmentation pathways with those of published data.

**Figure 8:**
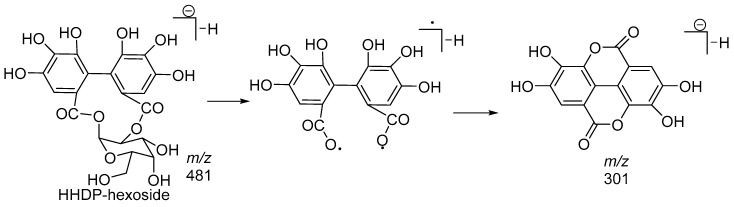
Fragmentation pathway of HHDP-hexoside.

Compound **14** showed the characteristic fragmentation pathway of an ellagitannin. The MS/MS spectrum showed ions at *m*/*z* 481 (loss of HHDP) and at *m*/*z* 301 (loss of HHDP-hexose). The fragmentation pathway of a galloyl-HHDP-hexose isomer is illustrated in Figure 8.

**Figure 9:**
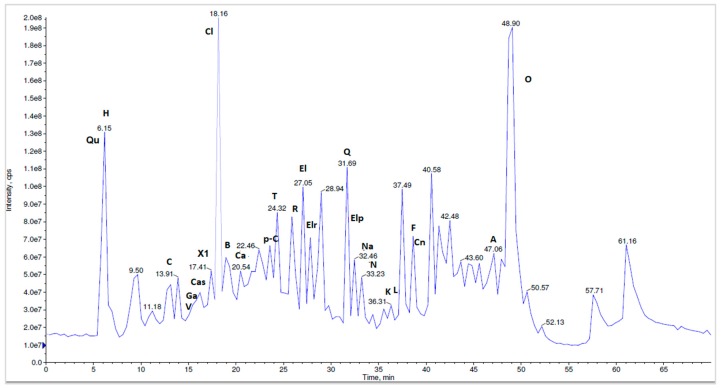
The LC-MS/MS chromatographic separation of the methanolic *N. alba* flower extract (H—HHDP-hexoside, Qu—quinic acid, C—corilagin, V—vanillic acid, Cas—castalin, Ga—gallic acid, X1—unidentified compound, Ca—caffeic acid, p-C—p-coumaric acid, T—tannic acid, R—rutin, El—ellagic acid, Elr—ellagic rhamnosyl acid, Elp—ellagic pentoside acid, Cn—cinnamic acid derivative, Na—naringenin, N—naringin, F—ferulic acid, Cl—chlorogenic acid, Q—quercetin, A—apigenin, L—luteolin, B—brevifolin, K—kaempferol, O—orientin).

**Figure 10:**
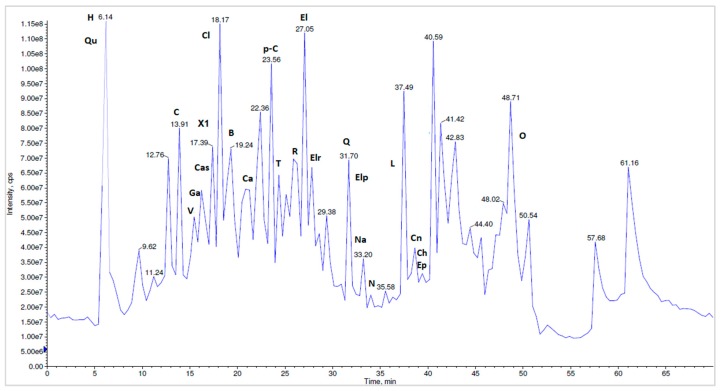
The LC-MS/MS chromatographic separation of the methanolic *N. alba* leaf extract (H—HHDP-hexoside, Qu—quinic acid, C—corilagin, V—vanillic acid, Cas—castalin, Ga—gallic acid, X1—unidentified compound, Ca—caffeic acid, p-C—p-coumaric acid, T—tannic acid, R—rutin, El—ellagic acid, Elr—ellagic rhamnosyl acid, Elp—ellagic pentoside acid, Cn—cinnamic acid derivative, Na—naringenin, N—naringin, Ch—catechin, Ep—epicatechin, Cl—chlorogenic acid, Q—quercetin, L—luteolin, B—brevifolin, O—orientin).

**Figure 11:**
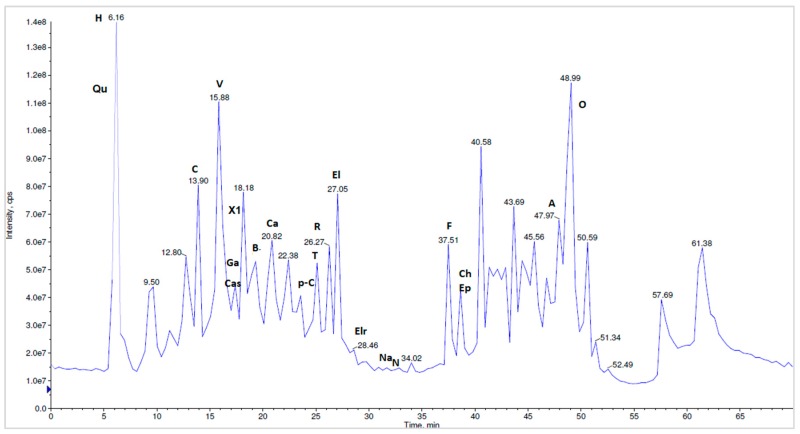
The LC-MS/MS chromatographic separation of the methanolic *N. alba* root extract (H—HHDP-hexoside, Qu—quinic acid, C—corilagin, V—vanillic acid, Cas—castalin, Ga—gallic acid, X1—unidentified compound, Ca—caffeic acid, p-C—p-coumaric acid, T—tannic acid, R—rutin, El—ellagic acid, Elr—ellagic rhamnosyl acid, Na—naringenin, N—naringin, F—ferulic acid, Ch—catechin, Ep—epicatechin, A—apigenin, B—brevifolin, O—orientin).

To the best of our knowledge, a total of 27 compounds are reported here for the first time as *N. alba* components from the Danube Delta landscape. The methanolic *N. alba* flower extract was separated by HPLC-MS/MS in the [M-H]^−^ mode. Overall, 25 different polyphenolic compounds (caffeic acid, p-coumaric acid, chlorogenic acid, naringin, naringenin, vanillic acid, quercetin, rutin, HHDP-hexoside, corilagin, tannic acid, gallic acid, ferulic acid, ellagic acid, ellagic acid pentoside, quinic acid, kaempferol, castalin, orientin, apigenin, luteolin, brevifolin, cinnamic acid derivative, ellagic acid rhamnosyl and another compound not yet identified) were separated and identified in the methanolic *N. alba* flower extract (Figure 9). In the *N. alba* leaf extract (Figure 10), 24 polyphenolic compounds were separated and identified. Compared with the flower extract, this extract contained the catechin (+) and epicatechin (−) isomers, which eluted together at a T_R_ of 39.67 min, with a [M-H]^−^ parent ion at 289 *m*/*z* and a fragment ion at 245 *m*/*z*, whereas kaempferol, apigenin, and ferulic acid were not identified. The stem extract contained 24 compounds that were identified and separated: caffeic acid, p-coumaric acid, catechin, epicatechin, naringin, naringenin, vanillic acid, corilagin, tannic acid, gallic acid, ferulic acid, ellagic acid, quinic acid, kaempferol, castalin, orientin, apigenin, luteolin, brevifolin, ellagic acid rhamnosyl, quercetin, rutin, HHDP-hexoside, and the compound that has not been identified yet. For the methanolic *N. alba* root extract (Figure 11), 21 out of the 27 polyphenolic compounds were identified; the missing ones were quercetin, chlorogenic acid, kaempferol, luteolin, ellagic acid pentoside, and the cinnamic acid derivative.

Table 3 contains a comparative presentation of the identified compounds in the methanolic extracts from the *N. alba* flower, leaf, stem, and root. The largest number of polyphenolic compounds was found in the flower extract, while the root extract contained the lowest number of polyphenolic compounds.

### 2.6. Macroelement and Microelement Contents

The bioavailability of macroelements and microelements depends on their nature and their association with the components in the soil. Plants easily assimilate macroelements and microelements through the roots. Other sources for these elements are rainfall, atmospheric dust, fertilizers, and chemicals that are used to protect plants that can be absorbed through the leaf blades. Plants used for therapeutic purposes must be collected from contamination-free areas. Regardless, as can be observed in the literature, the raw plant medical materials significantly differ with regards to their macroelement and microelement content. This content represents one of the criteria that have to be met in order for raw plant material to be used for medical purposes [25]. 

The positive effects of plant-based medicines were attributed to the presence of polyphenols and flavonoids, and can be correlated with their antioxidant activity, but they are also influenced by other organic and inorganic substances (macroelements and microelements). The macroelements and microelements play an important role as cofactors for various enzymatic systems and the augmentation of antioxidant components, or possess regulatory activity in the absorption and transport of water [26]. Between the antioxidant minerals, zinc is an essential component of the thymic hormone that is involved in immune cell production and controls and facilitates the maturation of lymphocytes, playing an important role in cell division [27]. Usually, research in this field only focuses on the organic components, but it is well known that some diseases require both macronutrients and micronutrients to be treated, and their effect depends on the dose at which they are administered. Relatively high quantities of macronutrients, such as calcium, phosphorus, sodium, potassium, and magnesium are necessary for a regular human diet [26].

The content of macroelements and microelements from the *N. alba* samples is shown in Table 4. Large quantities of potassium (4931.32–10,724.93 mg/kg), magnesium (1954.15–4084.08 mg/kg), phosphorus (1762.59–5181.95 mg/kg), and sodium (4692.36–36,712.48 mg/kg) were found in all four samples. While the root sample contained the largest quantities of magnesium (4692.36–36,712.48 mg/kg) and calcium (8621.94 mg/kg), the flower sample had the highest content of potassium (10,724.93 mg/kg) and phosphorus (5181.95 mg/kg), and the stem sample contained the largest amount of sodium (36,712.48 mg/kg).

Micronutrients such as iron, zinc, manganese, copper, molybdenum, cobalt, and chromium are present in the human body in very small quantities; however, unlike the other nutritive substances, they are essential to our survival [26]. The cobalt and molybdenum concentrations were under the detection limit in all of the *N. alba* samples. The chromium content varied from <1.0 to 14.56 mg/kg. The concentration of other microelements (Al, As, B, Ba, Cd, Hg, Li, Pb, Se, Sn, Ni) was determined as well, and most concentrations were under the detection limit. The lead content varied from <0.5 to 2.81 mg/kg, while all of the samples had a mercury and cadmium content that was under 0.5 mg/kg and 1.0 mg/kg, respectively. The lithium content was very small, varying between <1.0 mg/kg and 6.46 mg/kg.

## 3. Materials and Methods

### 3.1. Plant Material

The collection of plant samples is an important step in their analysis. The *N. alba* samples were collected in June 2017 from the Somava-Parcheș Lagoon Complex situated in the Danube Delta Biosphere Reserve. Specimens were deposited at the Botanical Garden of Galati, Romania.

### 3.2. Extraction

In order to extract the bioactive compounds, five dry samples of *N. alba* fruit, flowers, leaves, stems, and roots were used. The basic procedures, such as preliminary washing with distilled water and ultrapure water, drying the plant product at room temperature (7–12 days) until it reached a constant weight, and grinding to granulometry lower than 2 mm prior to extraction in order to obtain a homogenous sample were followed. The quantity of the samples was 10 g, and the main solvent that was used for the extraction of bioactive compounds was methanol (100 mL). Since the target compounds can be both polar and non-polar as well as thermally unstable, the extraction methods must be adapted for this case. The extracts were obtained by ultrasonication (35 Hz) for 2 h at 55 °C.

### 3.3. Microplate Determination of Total Polyphenol Content

The total polyphenol content was determined by applying the Folin–Ciocalteu method [17] to a 96-well plate analysis. First, 25 µL of Folin–Ciocalteu reagent was added to 10 µL of each methanolic extract. After 5 min of incubation, 25 µL of a 20% aqueous sodium carbonate solution and ultrapure water was added until the final volume reached 200 µL. Blanks were also prepared for each sample by replacing the Folin–Ciocalteu reagent with ultrapure water. A freshly prepared gallic acid solution (500–0.97 μg/mL) was used as a standard reference, and the results are given in equivalents of gallic acid per 1 g of sample. After 30 min, the absorbance values of the samples at 760 nm were recorded using a multiwell plate reader (Tecan Pro 200, Tecan Trading AG, Männedorf, Switzerland).

### 3.4. Microplate Determination of Total Flavonoid Content

The total flavonoid content was quantified by a 96-well plate analysis using aluminum chloride. First, 100 µL of an aqueous 2% aluminum chloride solution was added to 100 µL of each methanolic extract sample. After 15 min of incubation, the sample absorbance values at 415 nm were read using the Tecan Pro 200 multiwell plate reader. Quercetin was used as a reference standard (40–0.078 μg/mL), and the results are given in equivalents of quercetin per 1 g of sample [28].

### 3.5. Microplate Determination of Total Condensed Tannins Content

For the determination of the total condensed tannins content, a 96-well plate adaptation of Zou’s method was used [29]. First, 150 µL of a 4% methanolic vanillin solution and 75 µL of a 36% concentrated solution of HCl were added to 10 µL of each methanolic extract sample. After 15 min, the absorbance values at 500 nm were read against a blank solution that replaced the extract sample with 10 µL of ultrapure water. Aqueous solutions of (±) catechin with concentrations ranging from 10 µg/mL to 100 µg/mL were used as standards, and the results are reported in milligrams of catechin per 1 g of sample (mg EqC/g).

### 3.6. Microplate Determination of Antioxidant Activity Using DPPH

The antioxidant activity has been reported in various manners, such as the percentage of utilized reagent and the percentage of oxidation inhibition. Quercetin was used as a reference standard for an easier way of measuring antioxidant activity [18]. The DPPH-free radical has a maximum absorption at 517 nm, which gives a purple color. The color shifts from purple to yellow, resulting in a reduced form of DPPH. Antioxidant compounds can be water-soluble, insoluble, or bound to the cell walls. As such, the efficiency of extraction is an important factor in quantifying a plant’s antioxidant activity [9]. In a 96-well plate, 100 µL samples of a 100 µg/mL DPPH solution were added to 100 µL of methanolic extract samples with a 200 µg/mL concentration. The resulting solutions were then homogenized and then left in the dark at room temperature for 20 min, 35 min, and 50 min. The absorbance values at 517 nm were recorded after each time period. The blank sample used was a 1:1 mixture of methanol and DPPH solution. A 40 µg/mL solution of quercetin was used as a reference standard. The DPPH inhibition percentage, which relates to the antioxidant activity of quercetin and the extracts, was calculated using the following formula: % inhibition = (Ablank –Asample)/Ablank ×100 [28]. The concentration of sample that was required to scavenge 50% of DPPH (IC_50_) was graphically determined by plotting the percentage of inhibition against concentration of the inhibitor. The antioxidant activity index (AAI) was calculated according to the following formula: $AAI\;=concentration\;of\;DPPH\frac{µ\frac{g}{\mathrm{mL}}}{IC50}\left(µ\frac{g}{\mathrm{mL}}\right).$ Thus, the AAI value is calculated, taking into consideration both the mass of DPPH and the mass of the sample tested in the reaction, resulting in a constant for each compound that is independent of the DPPH concentration used in the sample [18].

### 3.7. HPLC-ESI-MS/MS Analysis of Polyphenolic Compounds

The HPLC-MS/MS analysis was carried out using a Q Trap 3200 Triple Quadrupole Mass spectrometer from Perkin Elmer (Waltham, MA, USA). In the chromatographic analysis, the Thermo C18 (150 × 4.6 mm, particle size of 5 μm) column (Thermo Fisher Scientific Inc., Waltham, MA, USA) was used with an injection volume of 25 µL. The solvents used were (A) formic acid (1%) and (B) methanol. The gradient elution was from 5% to 100% B at 30 °C, and the elution flow was set at 500 μL/min. Modifying the mobile phase’s pH value caused a significant change in the resolution of our compounds, especially for the phenolic acids. Formic acid is volatile, and is thus compatible with the LC-MS system. For the mass spectra, the ionization source temperature was 500 °C, and the spectra were recorded in the negative ion mode between 50 *m*/*z* and 650 *m*/*z* using argon as a collision gas. The pressure of the gas flux to the nebulizer was set at 1000 psi.

### 3.8. Determination of Macroelement and Microelement Contents

Due to the importance of the minerals and oligoelements present in medicinal plants [25], the quantities of macroelements and microelements from the *N. alba* extracts were experimentally determined. The amount of the following 23 elements was determined in three parallel measurements. For Al, B, Ba, Ca, Co, Cr, Cu, Fe, K, Li, Mg, Mn, Mo, Na, Ni, P, Zn, and Sn, the inductively coupled plasma optical emission spectrometer, Optima 8300DV (ICP-OES) from Perkin Elmer was used. For Hg, the atomic absorption spectrophotometer (AAS) PinAAcle 900T FIAS FLAME from Perkin Elmer was used, and because As, Cd, Pb, and Se showed quantities smaller than the detection limit, the atomic absorption spectrophotometer PinAAcle 900Z equipped with a longitudinally heated graphite oven from Perkin Elmer was used instead. The general detection limit was equal to the stock values from the Perkin Elmer machines. The standard solutions were prepared from the Merck standards (ICP, and AAS) and are found in the same matrix as in the samples. Sampling: 1 g from each *N. alba* sample with 8 mL of p.a. HNO_3_ and 2 mL of concentrated HCl were introduced in the quartz flasks of the Anton Paar oven. After mineralization, ultrapure water was added to the samples until a volume of 25 mL.

### 3.9. Statistical Analysis

All of the data were expressed as means ± standard deviation of the mean of three independent assays and analyzed through an unpaired Student’s *t*-test. Correlation between the data was carried out using the Microsoft Excel program.

## 4. Conclusions

To the best of our knowledge, this is the first comparative study that presents an exhaustive survey on the extraction, separation, identification, and analysis of the bioactive compounds of all of the anatomic parts of *N. alba* (fruit, flower, leaf, stem, and root) from the Danube Delta Biosphere Reserve. The evaluation of the bioactive compounds has been made using both qualitative and quantitative methods of analysis. The HPLC-MS/MS analysis highlighted the presence of polyphenolic and flavonoid compounds with antioxidant activities. The quantitative analysis included spectrophotometric determinations in order to evaluate the polyphenols, flavonoids, tannins, condensed tannins, and antioxidant activity. The antioxidant activity of the methanolic *N. alba* extracts, which were evaluated by using DPPH assay, is comparable with literature data gained through different techniques. The focus of antioxidant studies in the *Nymphaeaceae* family has been mainly on leaves and fruit, and any data from Romanian *N. alba* extracts has not been reported.

Samples from aquatic plants usually contain many different types of bioactive compounds. In the present study, we have proven that the ultrasound-mediated extracts of *N. alba* from the Danube Delta Biosphere Reserve had a high total polyphenol and flavonoid content. As secondary metabolites, polyphenols and flavonoids are strongly influenced by ecological stress, such as water deficit [10]. The total content of polyphenols and flavonoids is also influenced by the activity of specific enzymes in *N. alba*, which depends on the genetic material of the plant.

The ICP OES method, which was applied for a screening analysis of macronutrients and micronutrients, also revealed the presence of K, P, Na, Ca, and Mg in all of the parts of the plant; these minerals are necessary in different enzymatic processes, thus supporting some of the known therapeutic properties of the species.

Our results highlight the valuable contribution to the knowledge about the bioactive properties of native *N. alba* species from the Danube Delta Biosphere Reserve.

Further studies (unpublished results) are conducted in order to characterize the content in bioactive compounds and demonstrate other biological properties of *N. alba* species from the Danube Delta Biosphere Reserve.

## Figures and Tables

**Table 1:** Identification of the total condensed tannin content in different extracts of *N. alba* and ± standard deviation (mg C/g).

Total Condensed Tannin Content	Part of Plant				
	mg EqC/g or Negative (−)				
	Fruit	Flower	Leaf	Stem	Root
**Methanolic extracts**	0.5 ± 0.0	-	0.01 ± 0.0	2.9 ± 0.1	1.4 ± 0.0

- unidentified.

**Table 2:** Peak assignments and tentative identification of the phytochemical compounds in the *N. alba* extracts by HPLC-MS/MS in the negative ion mode.

No.	Compounds	T_R_^a^ (min)	Formula	[M-H]^− b^ *m*/*z*		Mass Error (ppm)	References
				Parent Ion	Fragment Ion (Relative Abundance %)		
1	HHDP ^c^-hexoside	6.15	-	481.06	301.14 (50), 463.20 (40)	−1.85	[2,15,19]
2	Quinic acid	6.16	C_7_H_12_O_6_	191.06	127.07 (33), 173.12 (50)	−2.21	[15]
3	Vanillic acid	15.35	C_8_H_8_O_4_	167.00	123.00 (30), 125.00 (100), 152.00 (10)	−0.91	[19]
4	Gallic acid	16.50	C_7_H_6_O_5_	169.00	125 (100)	−3.42	[15,19,20]
5	Castalin	16.93	C_27_H_20_O_18_	631.06	301.29 (100), 299.12 (37)	−1.52	[2]
6	X1 ^d^	17.29	-	-	613.20 (50), 301.13 (100), 631.15 (30)	−2.03	-
7	Chlorogenic acid	18.05	C_16_H_18_O_9_	355.00	163.00 (70)	−4.52	[20]
8	Corilagin	18.17	C_27_H_22_O_18_	633.07	301.14 (100), 589.22 (10)	−0.76	2
9	Brevifolin	19.53	C_10_H_12_O_4_	247.02	203.11 (75), 175.13 (20)	−4.28	[2,15]
10	Caffeic acid	20.37	C_9_H_8_O_4_	181.00	163.00 (95)	−3.98	[20]
11	p-Coumaric acid	23.85	C_9_H_8_O_3_	163.00	145.00 (30), 119.00 (10), 103.00 (30), 89.00 (10), 127.00 (8)	−1.96	[21]
12	Tannic acid	24.61	C_76_H_52_O_46_	183.50	123.70 (100)	−2.45	[22]
13	Rutin	26.16	C_27_H_30_O_16_	609.20	301.00 (100)	−2.5	[20]
14	Ellagic acid	27.31	C_14_H_6_O_8_	301.15	257.22 (100), 229.10 (50)	−0.1	[15,19]
15	Ellagic acid rhamnosyl	27.80	C_20_H_16_O_12_	447.02	359.17 (50), 403.20 (30), 385.11 (10), 315.25 (5), 301.13 (7), 275.18 (100)	−0.52	[2]
16	Quercetin	31.94	C_15_H_10_O_7_	301.00	151.00 (70)	−0.37	[20]
17	Ellagic acid-pentoside	32.07	C_19_H_14_O_12_	433.00	291.15 (21), 405.17 (57), 301.18 (90), 275.23 (8), 247.00 (40), 229.00 (5)	−2.51	[2,19]
18	Naringenin	32.46	C_15_H_12_O_5_	271.00	151.00 (75), 177.00 (100), 165.00 (52), 107.00 (18), 125.00 (25)	−1.98	[19]
19	Naringin	34.30	C_27_H_32_O_14_	579.00	151.00 (50), 119.00 (6), 271.00 (100)	−2.68	[23]
20	Kaempferol	36.60	C_15_H_10_O_6_	285.19	241.16 (67), 217.25 (100)	−3.98	[15]
21	Luteolin	36.92	C_15_H_10_O_6_	285.04	257.16 (40), 241.13 (100)	−4.58	[2,20]
22	Ferulic acid	38.09	C_10_H_10_O_4_	193.00	177.00 (7), 149.00 (50), 145.00 (100), 117.00 (32), 89.00 (62)	−2.98	[21]
23	Cinnamic acid derivative	38.65	-	329.09	197.10 (50), 239.10 (35), 169.07 (100)	−0.58	[2]
24	Catechin (+)	39.67	C_15_H_14_O_6_x H_2_O	289.00	245.00 (50), 205.00 (100), 179.00 (20), 261.00 (42)	−0.37	[15,19]
25	Epicatechin (−)	39.67	C_15_H_14_O_6_	289.00	245.00 (50), 205.00 (100), 179.00 (20), 261.00 (40)	−2.63	[15,19]
26	Apigenin	47.80	C_15_H_10_O_5_	269.03	223.01 (50), 179.07 (100)	−3.04	[2,20]
27	Orientin	49.74	C_21_H_20_O_11_	447.02	403.16 (100), 233.03 (50)	−1.61	[2]

^a^ TR—Retention time; ^b^ [M-H]^−^—Negative Ionization Mass/Mass spectrometry; ^c^ HHDP—Hexahydroxydiphenic acid; ^d^ X1—Unidentified yet.

**Table 3:** Polyphenols present in different *N. alba* extracts.

Polyphenols	Part of Plant			
	Positive (+) or Negative (−)			
	Flower	Leaf	Stem	Root
HHDP-hexoside	+	+	+	+
Catechin (+)	-	+	+	+
Chlorogenic acid	+	+	-	-
Corilagin	+	+	+	+
Vanillic acid	+	+	+	+
Caffeic acid	+	+	+	+
Tannic acid	+	+	+	+
Gallic acid	+	+	+	+
Epicatechin (−)	-	+	+	+
p-coumaric acid	+	+	+	+
Naringenin	+	+	+	+
Naringin	+	+	+	+
Rutin	+	+	+	+
Quercetin	+	+	+	-
Kaempferol	+	-	+	-
Quinic acid	+	+	+	+
Ellagic acid	+	+	+	+
Castalin	+	+	+	+
Orientin	+	+	+	+
Apigenin	+	-	+	+
Luteolin	+	+	+	-
Brevifolin	+	+	+	+
Ferulic acid	+	-	+	+
Ellagic acid-pentoside	+	+	-	-
X1	+	+	+	+
Ellagic acid rhamnosyl	+	+	+	+
Cinnamic acid derivative	+	+	-	-

- unidentified; + identified.

**Table 4:** Concentration of different elements in the dry residue of *N. alba* after ultrasonic extraction and ± standard deviation (mg/kg).

Elements	Part of Plant			
	mg/kg (Dry Weight)			
	Flower	Leaf	Stem	Root
**Al**	646.7 ± 15.2	1075.8 ± 57.8	224.9 ± 12.1	7151.9 ± 57.9
**As**	<1.0	<1.0	<0.5	<2.5
**B**	36.3 ± 1.3	32.2 ± 1.1	23.1 ± 1.2	<2.5
**Ba**	<2.5	13.5 ± 0.8	5.7 ± 1.0	55.1 ± 2.2
**Ca**	3817.1 ± 69.8	8103.1 ± 58.9	729.0 ± 38.0	8621.9 ± 113.3
**Cd**	<0.1	<0.1	<0.1	<0.1
**Co**	<1.0	<1.0	<1.0	2.0 ± 0.1
**Cr**	<1.0	<2.5	<1.0	14.5 ± 0.5
**Cu**	6.3 ± 0.5	3.0 ± 0.3	<1.0	10.2 ± 1.1
**Fe**	149.2 ± 6.6	817.0 ± 4.0	24.5 ± 1.8	539.6 ± 20.5
**Hg**	<0.5	<0.5	<0.5	<0.5
**K**	10,724.9 ± 45.2	4931.3 ± 17.7	6876.6 ± 19.9	7453.4 ± 18.8
**Li**	<1.0	<1.0	<1.0	6.4 ± 0.5
**Mg**	2857.2 ± 82.7	2057.3 ± 68.5	1954.1 ± 24.9	4084.1 ± 36.3
**Mn**	69.2 ± 1.7	508.3 ± 14.6	345.8 ± 6.3	379.0 ± 6.9
**Mo**	<1.0	<1.0	<1.0	<1.0
**Na**	9745.8 ± 55.9	16,908.6 ± 580.2	36,712.4 ± 589.1	4692.3 ± 67.7
**Ni**	<1.0	<2.5	<1.0	7.5 ± 0.5
**P**	5181.9 ± 57.2	2051.3 ± 34.3	1762.6 ± 14.7	3467.9 ± 23.8
**Pb**	<0.5	<0.5	<0.5	2.8 ± 0.3
**Zn**	64.1 ± 2.4	16.1 ± 1.8	12.7 ± 1.0	44.8 ± 2.9
**Se**	<1.0	<0.5	<0.5	<0.5
**Sn**	<2.5	<2.5	<2.5	<2.5
